# One‐year COVID‐19 outcomes on the oncology care patient pathway: Results of a French descriptive, cross‐sectional comprehensive study (ONCOCARE‐COV)

**DOI:** 10.1002/cam4.4817

**Published:** 2022-05-20

**Authors:** Léonard Laurent, Mathias Brugel, Claire Carlier, Florentin Clere, Aurélie Bertrand, Damien Botsen, Camille Boulagnon‐Rombi, Véronique Dalstein, Adeline Debreuve‐Theresette, Sophie Deguelte, Christian Garbar, Rachid Mahmoudi, Antonin Marechal, David Morland, Jean‐Baptiste Rey, Claire Schvartz, Catherine Vallet, Yacine Merrouche, Florian Slimano, Olivier Bouché

**Affiliations:** ^1^ Medical Oncology Department Godinot Cancer Institute Reims France; ^2^ Ambulatory Oncology Care Unit Reims University Hospital Reims France; ^3^ Care‐Associated Risks and Care Quality Department University Hospital Reims France; ^4^ Marne Site, Regional Coordination Center for Cancer Screening Grand‐Est Reims France; ^5^ Pathology Department Reims University Hospital Reims France; ^6^ INSERM, P3 Cell UMR‐S1250, SFR CAP‐SANTE Université de Reims Champagne‐Ardenne Reims France; ^7^ Medical Information Department Godinot Cancer Institute Reims France; ^8^ Surgery Department Reims University Hospital Reims France; ^9^ Pathology Department Godinot Cancer Institute Reims France; ^10^ Department of Internal Medicine and Geriatrics Reims University Hospital Reims France; ^11^ Université de Reims Champagne‐Ardenne Reims France; ^12^ Pharmacy Department Reims University Hospital Reims France; ^13^ Nuclear Medicine Department Godinot Cancer Institute Reims France; ^14^ CReSTIC EA 3804 Université de Reims Champagne‐Ardenne Reims France; ^15^ Pharmacy Department Godinot Cancer Institute Reims France; ^16^ Medical Information Department Reims University Hospital Reims France

**Keywords:** backlog, cancer care pathway, COVID‐19 pandemic, lockdowns

## Abstract

**Background:**

The COVID‐19 pandemic led to a widely documented disruption in cancer care pathway. Since a resurgence of the pandemic was expected after the first lockdown in France, the global impact on the cancer care pathway over the year 2020 was investigated.

**Aims:**

This study aimed to describe the changes in the oncology care pathway for cancer screening, diagnosis, assessment, diagnosis annoucement procedure and treatment over a one‐year period.

**Materials & Methods:**

The ONCOCARE‐COV study was a comprehensive, retrospective, descriptive, and cross‐sectional study comparing the years 2019 and 2020. All key indicators along the cancer care pathway assessing the oncological activity over four periods were described. This study was set in a high‐volume, public, single tertiary care center divided in two complementary sites (Reims University Hospital and Godinot Cancer Institute, Reims, France) which was located in a high COVID‐19 incidence area during both peaks of the outbreak.

**Results:**

A total of 26,566 patient's files were active during the year 2020. Breast screening (−19.5%), announcement dedicated consultations (−9.2%), Intravenous and Hyperthermic Intraoperative Intraperitoneal Chemotherapy (HIPECs) (−25%), and oncogeriatric evaluations (−14.8%) were heavily disrupted in regard to 2020 activity. We identified a clear second outbreak wave impact on medical announcement procedures (October, −14.4%), radiotherapy sessions (October, −16%), number of new health record discussed in multidisciplinary tumor board meeting (November, −14.6%) and HIPECs (November, −100%). Moreover, 2020 cancer care activity stagnated compared to 2019.

**Discussion:**

The oncological care pathway was heavily disrupted during the first and second peaks of the COVID‐19 outbreak. Between lockdowns, we observed a remarkable but non‐compensatory recovery as well as a lesser impact from the pandemic resurgence. However, in absence of an increase in activity, a backlog persisted.

**Conclusion:**

Public health efforts are needed to deal with the consequences of the COVID‐19 pandemic on the oncology care pathway.

## Graphical abstract

Key steps in the oncology care pathway activity were retrospectively analyzed and compared in this comprehensive and cross‐sectional study, between the different time settings in the COVID‐19 pandemic. Oncology care were heavily disrupted during the first and second COVID‐19 outbreak waves with decrease in screening and diagnosis activity as well as an uncommon stagnation in treatment activity. A backlog has inevitably persisted after the disruptions caused by the first and second outbreak waves even though a between‐lockdown recovery occurred in between. Public health efforts are needed to deal with the consequences

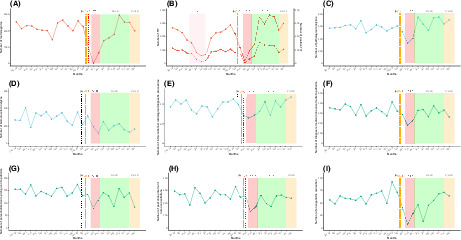

## 1 INTRODUCTION

Severe Acute Respiratory Syndrome Corona Virus 2 (SARS‐COV‐2) has spread globally since December 2019 causing a pandemic.^1^ On July 20, 2021, the pandemic was responsible for more than 112,000 deaths in France alone^2^ and the health care system was comprehensively reorganized, particularly with the first COVID‐19 outbreak wave which stressed healthcare systems with unprecedented organizational challenges.

Emergency care and intensive care units (ICUs) were prioritized, albeit sometimes to the detriment of treating other serious conditions such as myocardial infarction and/or general admissions for chronic diseases.^3^, ^4^, ^5^ The World Health Organization (WHO) reported that one in three European countries experienced cancer care disruptions during the early period of the pandemic.^5^, ^6^

In cancer care, scientific organizations have provided guidelines designed to prevent healthcare reorganization from negatively affecting cancer screening, diagnosis, and treatments.^7^, ^8^, ^9^, ^10^ Consequences of the first outbreak wave on the oncology care pathway were described in the preliminary findings of the French ONCOCARE‐COV study.^11^ Results confirmed a dramatic decrease during the first lockdown in each different aspect of a patient's cancer care pathway, namely in breast and colon cancer screening (−100%), histopathological activities (−48%), oncology announcement consultations (−49%), and new patient medical records discussed in Multidisciplinary Tumor Board Meeting(s) (MTBM) (−39%). Moreover, cancer treatments, oncological surgical activity had the greatest fall (−30%), whereas a lesser impact was observed on antineoplastic agent preparation (−9%) and radiotherapy sessions (−16%).^11^ This downward trend was experienced in other European countries including the Netherlands and Spain. It was also confirmed in a systemic review that included 62 studies from all over the world.^12^, ^13^, ^14^

Despite better organization, the second wave of the COVID‐19 pandemic continued to disrupt healthcare systems. Repeated local and national outbreaks may have had impacts over the subsequent months raising questions about a rebound in oncology care activity. However, describing the pandemic's impact on cancer care pathway over the year 2020 has remained limited since most studies have only focused on the first wave disruptions. This study aimed to describe the changes in the oncology care pathway for cancer screening, diagnosis, assessment, diagnosis announcement procedure, and treatment over a 1‐year period.

## 2 PARTICIPANTS AND METHODS

### 2.1 Study design

A retrospective, descriptive, comprehensive, and cross‐sectional study were performed from January 01, 2019 to December 31, 2020 in one area of France (Marne) with high COVID‐19 incidence rates (*Région Grand‐Est*). Diagnosis and treatment activity were collected in a single public tertiary care center divided into two complementary sites (Reims University Hospital, Reims France and Godinot Cancer Institute, Reims, France). Screening activity was collected at a regional level from the regional screening center (Regional Coordination Center for Cancer Screening: Marne/*Région Grand‐Est*).

Four time periods were defined based on the French governmental containment measures initiated for COVID‐19. The first period was defined as the pre‐lockdown (January 01, 2019 to March 17, 2020) which corresponded to the usual level of activity seen before the pandemic. A second period was defined as the first lockdown (1st LD) and lasted from March 17, 2020 to May 11, 2020. The third period occurred between lockdowns from May 11, 2020 to October 31, 2020 and lastly, a second lockdown period (2nd LD) from October 30, 2020 and continued until the end of the year.

### 2.2 Patients

All screening, diagnosis, and treatment activities were performed on adults over 18 years old either suffering from or free of a malignant disease (solid tumor and hematologic malignancies). All participants had to be in contact with the oncological care pathway (screening, diagnosis, assessment, or treatment) at some point.

### 2.3 Data collection

Indicators were chosen at specific key steps all along the pathway for screening, diagnosis, assessment, diagnosis announcement procedure, and treatment. Screening was evaluated through the monthly number of mammograms, FITs (Fecal Immunological Test), both in number and positivity rate using data compiled from the regional screening center (Regional Coordination Center for Cancer Screening: Marne/*Région Grand‐Est*). Monthly oncological activity indicators were gathered using electronic medical record data from the Cancer Coordination Center (3C) and nationwide procedure codes (*Classification Commune des Actes Médicaux*, CCAM). Diagnosis activity was assessed with the number of histopathological analyses (cytology, biopsy, surgical specimens, and extemporaneous analyses), interventional radiology diagnostic procedures, and 18F‐fluorodeoxyglucose positron emission tomography coupled with computer tomography (CT). Monthly overall and first‐time discussed medical records in MTBMs at each of the two cancer centers were numbered. Moreover, we analyzed cancer announcement modalities (number of medical announcement consultations), given personalized care plans, and post diagnosis nurse consultations.^15^

The number of initial oncogeriatric assessments was also collected. Medical treatment activity was assessed by the number of venous access devices implanted, chemotherapy units prepared known as the Intravenous and Hyperthermic Intraoperative Intraperitoneal Chemotherapy (HIPEC), outpatient cancer care unit admissions, occupation rates, and active lists. Radiotherapy activity was assessed by the number of radiotherapy sessions and CT simulation sessions that preceded them and the active list of radiotherapy patients. The number of performed carcinological surgeries and interventional therapeutic radiological procedures (hepatic embolization and thermo‐ablative procedures) were used to quantify interventional treatment activity.

The weekly average number of COVID‐19 patient admissions in medical units and ICUs (Reims University Hospital only) were used to assess the local impact of the pandemic in addition to the cumulative number of deaths due to COVID‐19.

### 2.4 Ethics

Since this study was non‐interventional, the approval by an independent ethical committee was not required. All data were extracted from CCAM and institutional data without patient health record reviews. The institutional review board at Reims University Hospital approved this study and the ONCOCARE‐COV study is registered on ClinicalTrials.gov (NCT04445870).

### 2.5 Statistical analysis

Quantitative data were numbered per week, month, and/or year depending on the variable. No qualitative data were used during this study nor were statistical tests performed. The number of monthly new patients or the average of oncology procedures throughout the study period were described and compared to the previous year as a monthly standardized rate: (2020 activity‐2019 activity)/(2019 activity). Previous year activity was considered as the baseline. Missing values were not included in percentage calculations. Trends were visually compared using temporal curves. All graphic representations and statistical analyses were performed using R (R development core team, Version 1.2.5019) and Microsoft Excel (Version 2018).

## 3 RESULTS

**FIGURE 1:**
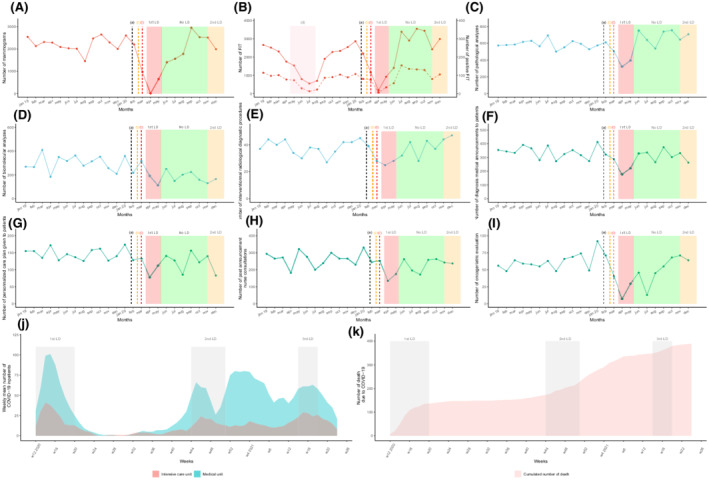
Temporal curves of monthly diagnostic and assessment oncological activity volume (between January 2019 and December 2020) at the Reims University Hospital and Cancer Institute (France), of weekly number of COVID‐19 inpatients. And cumulated number of death due to COVID‐19. The plain curve represents the number of procedures per month between January 2019 and December 2020. The dashed curve in B represents the number of positive FIT. (A) Number of mammograms performed in Marne département. (B) Number of FIT performed in Marne département. (C) Number of overall carcinologic histopathological analyses. (D) Number of overall biomolecular analyses (somatic and germline). (E) Number of interventional radiology diagnostic procedures performed. (F) Number of medical diagnostic announcement. (G) Number of personalized care plan given to patients. (H) Number of post announcement nurse consultations. (I) Number of oncogeriatric evaluations. (J) Mean weekly number of COVID‐19 inpatients. (K) Cumulated number of death due to COVID‐19. (a) The black dashed vertical line marks timeline of first diagnosed COVID‐19 patient in France (January 24, 2020). (b) The orange dashed vertical line marks timeline of first COVID‐19 deceased patient in France (February 15, 2020). (c) The red dashed vertical line marks timeline of first COVID‐19 admitted patient in Reims (February 27, 2020). (d) The red area marks a period of FIT stock shortage (from April 15, 2019 to July 25, 2019). The red area represents the first lockdown period from March 17 to May 11, 2020. The green area represents the period without lockdown from May 11 to October 31, 2020. The orange area represents the second lockdown period from October 31, 2020 to June 30, 2021. COVID‐19, coronavirus disease 2019; FIT, fecal immunochemical test; LD, lockdown; MTBM, multidisciplinary tumor board meetings; w, weeks.

Between 2019 and 2020, the volume of screening activity for the Marne area totaled 47,457 mammograms and 49,216 FITs. The two centers reviewed 24,134 files in MTBMs including 11,649 for first‐time patients (Table 1). Treatment activity included 5882 oncological surgeries, 1338 catheter implantations, 51,783 radiotherapy sessions, and 42,734 outpatient care unit admissions for intravenous chemotherapy treatment. From March 01, 2020 to May 30, 2021, COVID‐19 caused 389 inpatient deaths (Figure 1J,K).

**TABLE 1:** Annual activity and monthly activity difference between 2019 and 2020 on essential oncology care pathway steps

	Total per year		Annual activity difference	Monthly activity difference between 2020 and 2019 (%)											
	2019	2020		January	February	March	April^a^	May	June	July	August	September	October	November^b^	December
Screening															
Mammograms	26,293	21,164	−19.5%	2.4%	3.6%	−58.1%	−99.6%	−68.9%	−31.0%	−22.5%	22.2%	19.2%	−4.6%	9.4%	−0.6%
FIT	21,847	27,369	25.3%	7.6%	−13.3%	−49.7%	−89.3%	−40.0%	77.0%	513.1%	315.3%	86.4%	51.1%	3.8%	17.3%
Positive FIT	895	1095	22.3%	−6.1%	−17.5%	−24.8%	−92.2%	−53.4%	96.6%	1084.6%	513.6%	53.5%	43.3%	−23.1%	18.0%
Ratio positive FIT/performed	41.5%*	37%*	−10.8%	−14.0%	−7.7%	6.8%	−43.2%	−29.2%	8.1%	95.8%	43.8%	−17.8%	−5.0%	−26.7%	0.0%
Diagnosis															
Histopathological diagnosis															
Biopsies	1659	1879	13.3%	14.4%	9.9%	3.9%	−27.2%	−31.7%	−0.6%	−31.6%	3.0%	70.7%	123.1%	25.2%	45.0%
Surgical specimens	2553	2589	1.4%	−7.9%	−13.9%	−11.2%	−35.0%	−42.6%	50.5%	4.0%	1.1%	25.4%	10.7%	12.4%	43.7%
Extemporaneous analyses	152	208	36.8%	41.7%	25.0%	20.0%	−23.1%	−71.4%	35.7%	85.7%	244.4%	42.9%	30.8%	26.7%	133.3%
Cytologies	2671	2498	−6.5%	−2.3%	19.3%	−28.6%	−69.7%	−31.0%	42.3%	−6.3%	5.3%	18.0%	−9.8%	−6.9%	13.7%
Overall histopathological analyses	7035	7174	2.0%	0.2%	5.5%	−14.0%	−47.9%	−37.2%	33.2%	−7.8%	7.4%	33.9%	20.8%	8.4%	33.9%
Other diagnosis procedures															
Diagnostic interventional radiology procedures	450	438	−2.7%	21.6%	−11.4%	−30.0%	−43.2%	−17.6%	6.7%	10.5%	−24.3%	59.3%	5.7%	4.8%	11.9%
PET/CT performed	4002	4363	9.0%	14.3%	7.6%	7.8%	−22.7%	16.0%	28.2%	4.4%	2.7%	12.7%	6.9%	20.3%	15.5%
Biomolecular analyses															
Constitutionnal															
Colorectal	14	10	−28.6%	^c^											
Pancreas	14	8	−42.9%												
Breast/ovary	441	184	−58.3%												
Others	254	170	−33.1%												
Total	723	374	−48.3%	22.4%	−40.0%	10.4%	192.9%	−77.8%	3.1%	−84.0%	−88.2%	−67.5%	^d^	^d^	^d^
Somatic															
Colorectal	341	190	−44.3%	^c^											
Lung	375	294	−21.6%												
Breast	58	94	62.1%												
Melanoma	125	103	−17.6%												
Others	136	185	36.0%												
Total	1088	877	−19.4%	35.6%	−6.7%	−44.2%	−34.2%	−60.4%	−34.4%	−38.8%	13.6%	12.3%	−16.7%	−16.3%	−1.2%
Overall biomolecular analyses	3569	2489	−30.3%	33.5%	−18.0%	−22.9%	6.0%	−67.1%	−20.5%	−58.4%	−26.7%	−28.0%	−55.0%	−49.4%	−21.1%
Assessment and diagnosis announcement procedure^e^															
Medical announcement consultations	4003	3633	−9.2%	16.3%	−6.4%	−14.1%	−53.8%	−38.7%	17.1%	−12.9%	−2.6%	15.4%	−14.4%	4.7%	−4.0%
Given personalized care plans	1741	1486	−14.6%	12.3%	−16.8%	−0.7%	−54.1%	−11.7%	−3.4%	−6.6%	−31.7%	−0.6%	−24.7%	10.2%	−40.7%
Post diagnosis nurse consultations	3113	2771	−11.0%	12.6%	−7.9%	−6.6%	−25.8%	−45.7%	−5.1%	−2.0%	−28.0%	−14.0%	−1.1%	−8.6%	3.0%
Oncogeriatric evaluations	709	604	−14.8%	64.3%	47.9%	−35.9%	−86.4%	−48.3%	−16.4%	−79.4%	−6.3%	−16.7%	−1.4%	−4.1%	30.6%
Treatment															
Implanted venous access devices	672	666	−0.9%	7.3%	14.0%	21.8%	−17.9%	−31.4%	11.3%	−3.0%	−13.6%	−9.8%	−9.5%	30.2%	−3.6%
Oncology daycare visits for chemotherapy	10,863	11,011	1.4%	15.3%	13.0%	6.4%	−6.4%	−7.0%	−1.1%	−5.0%	1.7%	6.1%	−6.4%	−1.8%	4.9%
Active medical oncology patients	913*	970*	6.2%	14.8%	11.6%	9.0%	−0.7%	2.1%	6.6%	1.9%	3.5%	5.7%	3.1%	11.3%	5.1%
Oncology daycare occupation rate	236.3%*	215.6%*	−8.7%	2.4%	−0.5%	−9.0%	−15.6%	−8.5%	−13.1%	−8.5%	−11.3%	−7.0%	−8.3%	−9.2%	−8.7%
Hyperthermic intraperitoneal chemotherapy bags	32	24	−25.0%	−20.0%	−83.3%	−100.0%	−100.0%	−100.0%	200.0%	66.7%	0.0%	300.0%	−50.0%	−100.0%	0.0%
Prepared chemotherapy bags	48,869	50,533	3.4%	19.9%	15.3%	8.0%	−1.4%	−11.7%	10.3%	−0.4%	−0.7%	7.4%	−7.0%	−1.8%	8.4%
Carcinologic surgery procedures	2969	2913	−1.9%	−11.6%	17.2%	1.8%	−29.1%	−20.6%	−8.2%	4.8%	5.4%	15.7%	−0.8%	7.0%	2.4%
Computed Tomography simulation sessions	1574	1510	−4.1%	−21.0%	−16.2%	−22.5%	4.3%	−28.6%	29.8%	−9.9%	4.0%	−16.8%	−2.8%	24.8%	27.4%
Radiotherapy courses	25,875	25,908	0.1%	−3.4%	−1.5%	14.5%	−15.6%	−7.9%	39.3%	4.1%	8.0%	−2.9%	−16.0%	−2.8%	−5.9%
Active radiotherapy patients	232*	227*	−2.2%	−5.2%	−5.0%	−0.4%	−0.9%	−4.2%	29.1%	5.2%	3.7%	−7.3%	−7.7%	−3.1%	2.8%
Therapeutic interventional radiology procedures	91	113	24.2%	350.0%	42.9%	−50.0%	−44.4%	300.0%	−16.7%	−27.3%	114.3%	28.6%	44.4%	50.0%	50.0%
Hepatic chemoembolizations	50	41	−18.0%	−28.6%	−33.3%	−25.0%	−50.0%	14.3%	0.0%	−100.0%	−20.0%	−66.7%	0.0%	150.0%	100.0%

*Note*: Monthly changes in volume of oncology activities (%) are calculated with (2020 activity – 2019 activity)/2019 activity and are illustrated through color variation from green (rising activity) to red (decreasing activity). Gray areas show unavailable data for the analyses. Data presented with (*) are annual medians.

Abbreviations: FIT, fecal immunochemical test; PET/CT, Positron emission tomography with computed tomography.

^a^ Overall inpatients peak at Reims (April 05, 2020).

^b^ Overall second inpatients peak at Reims (November 15, 2020).

^c^ Detailed data are not presented due to futility by low number of patients per month.

^d^ Constitutional biomolecular activity was gradually transferred in another center *from July 01, 2020*.

^e^ Diagnosis announcement procedure (three steps) is a measure of the first French cancer plan (2003–2007).

Breast cancer screening activity fell from 26,293 to 21,164 mammograms between 2019 and 2020 (−19.5%) (Table 1 and Figure 1A,B). Colorectal cancer (CRC) screening increased by 25.3% rising from 21,847 to 27,369 FITs from 2019 to 2020. FIT positivity rates dropped by 10.8% in 2020 compared with 2019. Screening activity dropped during March, April, and May 2020 by 58.1%, 99.6%, and 68.9%, respectively, and by 49.7%, 89.3%, and 40% for breast cancer and colorectal screening, respectively. Screening activity increased considerably during the months between lockdowns. However, during the second lockdown period, no significant decline was observed. The number of screening procedures performed remained at or above the previous year's activity in November (+9.4% for mammograms and +3.8% for FITs) and December (−0.6% for mammograms and +17.3% for FITs). The number of positive FITs and the corresponding positivity rate varied according to the number of analyzed tests. We observed a 12‐fold increase in positive FITs in May 2020 compared with 2019. However, it is important to emphasize that from April to August 2019, the screening campaign was affected by an FIT supply shortage.

The number of cytological, biopsy, surgical specimen, and extemporaneous analyses declined in 2020 with a drop during the first pandemic peak (−23.1% to −71.4%) compared to the previous year (Table 1 and Figure 1C–I). All monthly histopathological analyses rose during the period from June to December without any observed impact from the second pandemic infection wave. Interventional radiology diagnostic procedures followed the same pattern.

The number of diagnosis announcement procedures were significantly lower during 2020 compared to 2019. The number of medical announcement consultations saw significant decreases from March to May 2020 (−14.1%; −53.8%; −38.7%, respectively). Similar trends in the number of given personalized care plans (−0.7%; −54.1%; −11.7%, respectively), post diagnosis nurse consultations (−6.6%; −25.8%; −45.7%, respectively), and oncogeriatric evaluations (−35.9%; −86.4%; −48.3%, respectively) were also observed. The anticipated recovery did not appear at the end of the first lockdown and no noticeable decrease was perceived during the second wave.

**FIGURE 2:**
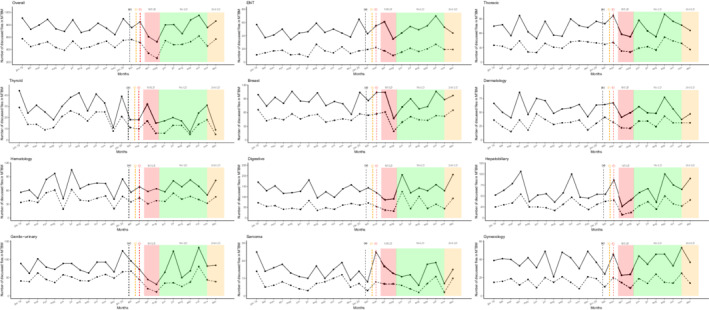
Temporal curves of monthly MTBM activity volume (between January 2019 and December 2020) at the Reims University Hospital and Cancer Institute (France). The plain curve represents the number of overall files presented per month from January 2019 to December 2020. The dashed curve represents the number of new patients' files presented in MTBM per month from January 2019 to December 2020. (a) The black dashed vertical line marks timeline of first diagnosed COVID‐19 patient in France (January 24, 2020). (b) The orange dashed vertical line marks timeline of first COVID‐19 deceased patient in France (February 15, 2020). (c) The red dashed vertical line marks timeline of first COVID‐19 admitted patient in Reims (February 27, 2020). The red area represents the first lockdown period from March 17 to May 11, 2020. The green area represents the period without lockdown from May 11 to October 31, 2020. The orange area represents the second lockdown period from October 31, 2020 and June 30, 2021. COVID‐19, coronavirus disease 2019; ENT, ear, nose and throat; LD, lockdown; MTBM, multidisciplinary tumor board meetings.

The number of health records discussed in the MTBMs remained almost stable between 2019 and 2020 (12,051 vs. 12,083, [+0.3%]) despite an unusual decrease in April and May (−36.0% and −26.3%) (Table 2 and Figure 2). Most organ specialized MTBMs experienced a decline in the total number of discussed health records in April and May compared to 2020 except for ENT (April, +22.0%), neurological malignancies (April, +65.6%), breast cancer (April, +30.0%), and thyroid malignancies (+28.0%). The most significant drops were detected for bone malignancies (April, −81.8%), neuroendocrinology (April, −80.4%), GIST (April, −80.0%), hepatobiliary (April, −74.5%), genitourinary (May, −56.2%), and breast cancer (May, −55.7%). Also, there were fewer newly diagnosed patient files discussed in April and May except for cases of neurological malignancies (+170%), breast cancer (+38.6%), thyroid malignancies (+88.9%), dermatological tumors (+16.7%), and sarcomas (+16.7%). There was an appreciable rise in the number of newly discussed records in the months of June and August after the first pandemic wave (+39.4% and 16.5%, respectively) which contrasted with a slight variation in the overall number (18.7% and −2.0%, also respectively). As the second wave of the pandemic unfolded (Figure 1), MTBM monthly activity rose from September to December 2020 regarding the total number of records discussed with an exception seen by an unusual drop in November (−14.6%) for first‐time discussed patient files during the second lockdown.

**TABLE 2:** Annual activity (number of overall and new discussed files) and monthly activity difference during 2019 and 2020 for MTBM

	Total per year		Annual activity difference	Monthly activity difference between 2020 and 2019 (%)											
	2019	2020		January	February	March	April^a^	May	June	July	August	September	October	November^b^	December
General description															
Overall	12,051	12,083	0.3%	−6.3%	2.6%	3.9%	−36.0%	−26.3%	18.7%	−10.2%	−2.0%	25.7%	11.5%	0.2%	35.0%
Total of new files	5730	5919	3.3%	−4.5%	24.9%	5.3%	−35.0%	−39.5%	39.4%	−10.6%	16.5%	19.2%	25.0%	−14.6%	31.0%
Overall number of files discussed															
Genito‐urinary	969	976	0.7%	39.3%	54.8%	−28.4%	−43.0%	−56.2%	−27.0%	38.2%	−29.6%	9.5%	43.0%	−11.8%	27.3%
Thoracic	850	906	6.6%	13.3%	2.6%	76.4%	−40.2%	−15.9%	77.6%	−23.5%	−5.4%	85.2%	−5.6%	1.3%	−8.3%
Bone	82	109	32.9%	225.0%	−16.7%	−12.5%	−81.8%	−50.0%	125.0%	300.0%	10.0%	−36.4%	200.0%	75.0%	100.0%
Dermatology	702	658	−6.3%	−4.5%	28.0%	67.5%	−52.3%	6.5%	−20.0%	−31.0%	0.0%	37.5%	−3.4%	−42.2%	11.9%
ENT	545	628	15.2%	14.0%	16.2%	34.1%	22.0%	2.9%	0.0%	17.4%	−16.9%	22.2%	40.0%	20.5%	15.8%
Hematology	817	821	0.5%	51.8%	−8.2%	58.1%	−31.0%	−34.0%	35.7%	−22.9%	6.3%	13.7%	−9.2%	−33.3%	100.0%
Neurology	327	372	13.8%	58.3%	7.1%	−12.5%	65.6%	15.4%	29.6%	−59.0%	11.1%	43.5%	3.4%	0.0%	40.0%
Digestive	1658	1716	3.5%	−27.8%	9.8%	−20.8%	−27.3%	−22.0%	62.8%	−29.3%	45.5%	7.1%	59.8%	−0.7%	26.6%
Neuroendocrinology	388	339	−12.6%	−20.0%	−29.4%	7.1%	−80.4%	−35.3%	−48.0%	105.9%	−51.5%	89.7%	−36.1%	16.7%	108.3%
Hepatobiliary	728	755	3.7%	3.8%	−14.3%	11.5%	−74.5%	13.5%	11.5%	21.8%	0.0%	75.4%	−25.0%	38.3%	100.0%
Sarcoma	177	165	−6.8%	−44.0%	−42.9%	56.3%	−5.6%	0.0%	0.0%	20.0%	−64.7%	80.0%	26.7%	−63.2%	66.7%
Gynecology	465	448	−3.7%	−5.1%	−41.5%	15.0%	−30.3%	−42.9%	41.9%	−28.6%	119.0%	−4.2%	−21.4%	76.7%	−24.5%
Breast	1017	1039	2.2%	5.1%	16.2%	11.8%	30.0%	−55.7%	−15.3%	8.4%	−34.0%	−2.7%	26.2%	11.4%	73.2%
GIST	53	60	13.2%	−66.7%	−25.0%	−14.3%	−80.0%	33.3%	33.3%	800.0%	−50.0%	200.0%	25.0%	400.0%	−33.3%
Thyroid	363	247	−32.0%	−11.4%	−25.0%	−41.9%	28.0%	−16.7%	−43.3%	−47.4%	−59.5%	−73.1%	−41.5%	−6.1%	−18.2%
Oncogenetic	150	58	−61.3%	^c^											
Thrombosis	24	10	−58.3%												
Biomolecular	17	26	52.9%												
Number of new files discussed															
Genito‐urinary	578	509	−11.9%	61.0%	74.4%	−28.6%	−55.6%	−70.3%	−39.7%	−30.8%	−38.1%	−7.3%	55.8%	−25.4%	−20.4%
Thoracic	406	409	0.7%	14.3%	12.1%	57.7%	−47.7%	0.0%	45.0%	−17.9%	−19.4%	79.3%	4.8%	−11.4%	−38.1%
Bone	60	90	50.0%	200.0%	0.0%	−20.0%	−80.0%	−50.0%	100.0%	1000.0%	33.3%	−22.2%	1100.0%	140.0%	71.4%
Dermatology	358	374	4.5%	−16.7%	70.8%	113.3%	−38.9%	16.7%	−27.7%	−8.1%	−14.3%	38.7%	−3.1%	−13.9%	72.2%
ENT	185	222	20.0%	54.5%	35.7%	29.4%	−5.6%	−9.1%	25.0%	162.5%	−40.7%	17.6%	92.9%	−17.4%	46.2%
Hematology	490	491	0.2%	38.2%	−7.9%	29.4%	−43.6%	−37.7%	89.5%	−12.9%	5.1%	27.0%	−7.0%	−33.3%	130.0%
Neurology	109	136	24.8%	10.0%	50.0%	−45.5%	170.0%	−26.7%	25.0%	−52.9%	18.2%	57.1%	175.0%	300.0%	0.0%
Digestive	678	803	18.4%	−18.4%	28.1%	−9.7%	−13.3%	−25.0%	204.9%	−39.8%	168.4%	−2.0%	52.3%	−22.2%	33.3%
Neuroendocrinology	107	138	29.0%	63.6%	55.6%	36.4%	−76.9%	−33.3%	−66.7%	^d^	−72.7%	75.0%	109.1%	−23.1%	175.0%
Hepatobiliary	360	366	1.7%	24.0%	26.7%	20.6%	−85.2%	−48.0%	12.0%	60.9%	17.6%	61.3%	−31.9%	−10.0%	115.8%
Sarcoma	88	74	−15.9%	−50.0%	−40.0%	33.3%	−12.5%	16.7%	50.0%	33.3%	−71.4%	−12.5%	10.0%	−83.3%	100.0%
Gynecology	189	189	0.0%	0.0%	0.0%	0.0%	0.0%	0.0%	0.0%	0.0%	0.0%	0.0%	0.0%	0.0%	0.0%
Breast	583	595	2.1%	−13.6%	28.6%	18.8%	38.6%	−66.7%	−15.2%	−3.8%	−21.4%	10.3%	23.3%	13.0%	51.2%
GIST	30	26	−13.3%	^c^											
Thyroid	222	139	−37.4%	−27.6%	−21.4%	−28.6%	88.9%	−45.5%	−71.4%	−50.0%	−43.5%	−70.6%	−44.0%	−28.0%	−37.5%
Oncogenetic	150	58	−61.3%	^c^											
Thrombosis	22	10	−54.5%												
Biomolecular	17	26	52.90%												

*Note*: Monthly changes in volume of oncology activities (%) are calculated with (2020 activity – 2019 activity)/2019 activity and are illustrated through color variation from green (rising activity) to red (decreasing activity). Gray areas show unavailable data for the analyses.

Abbreviations: ENT, ear nose and throat; GIST gastro intestinal stromal tumor; MTBM, multidisciplinary tumor board meeting; NA, not applicable.

^a^ Overall first inpatients peak at Reims (April 05, 2020).

^b^ Overall second inpatients peak at Reims (November 15, 2020).

^c^ Detailed data are not presented due to futility by low number of patients per month.

^d^ No files were presented in July 2019.

**FIGURE 3:**
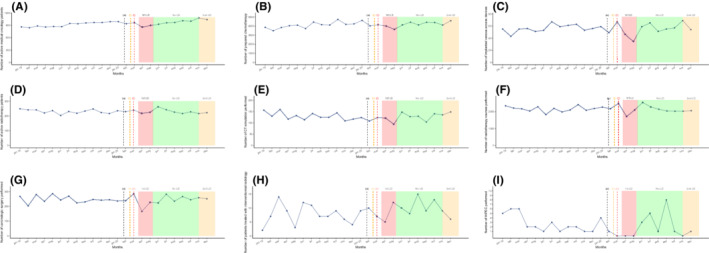
Temporal curves of monthly therapeutic activity volume (between January 2019 and December 2020) at the Reims University Hospital and Cancer Institute (France). (a) The black dashed vertical line marks timeline of first diagnosed COVID‐19 patient in France (January 24, 2020). (b) The orange dashed vertical line marks timeline of first COVID‐19 deceased patient in France (February 15, 2020). (c) The red dashed vertical line marks timeline of first COVID‐19 admitted patient in Reims (February 27, 2020). (A) Number of active medical oncology patients. (B) Number of prepared chemotherapy units. (C) Number of implanted venous access devices. (D) Number of active radiotherapy patients. (E) Number of CT simulation sessions. (F) Number of radiotherapy courses performed. (G) Number of carcinologic surgeries performed. (H) Number of therapeutic interventional radiology procedures performed. (I) Number of hyperthermic intraperitoneal chemotherapy performed. The red area represents the first lockdown period from March 17 to May 11, 2020. The green area represents the period without lockdown from May 11 to October 31, 2020. The orange area represents the second lockdown period from October 31, 2020 and still ongoing. LD, lockdown; COVID‐19, coronavirus disease 2019.

The number of implanted venous catheters, prepared chemotherapy units, active medical oncology patients, and outpatient care unit admissions experienced a moderate decrease during the first lockdown peak of around 10%–15% (Table 1 and Figure 3). However, the number of prepared HIPEC plummeted from March to May 2020 (−100%). All chemotherapy preparation activity remained globally stable in the months following the first outbreak wave compared to 2019 and did not seem to be disrupted during the second lockdown.

The number of radiotherapy sessions decreased in April and May (−15.6% and −7.9%, respectively) and CT simulation sessions also fell (May, −28.6%). The number of radiotherapy sessions rose in June 2020 following the first lockdown (+39.3%). The second lockdown period only moderately impacted the number of radiotherapy sessions with a single month decrease in October (−16.1%) which was preceded by a drop in CT simulation sessions in September (−16.8%). Concerning medical oncology and radiotherapy activity, we did not identify any tangible recovery during the months following the first lockdown.

Interventional radiology therapeutic procedures (hepatic embolization and thermo‐destruction) dropped during the first lockdown (March, −50%; April, −44.4%). The number of procedures subsequently skyrocketed in June but experienced a slight decline in July (−16.7%) and August (−27.3%). The second lockdown period did not seem to affect this activity.

The total number of surgical procedures globally declined between March and May with April seeing a decrease of 29.1%. A constant increase was observed from July to September (4.8%, 5.4%, and 15.7%, respectively) with overall activity that was similar when compared to the previous year. Surgery activity during the second pandemic peak remained the same as in 2019.

## 4 DISCUSSION

This study was a retrospective, descriptive, and comprehensive overview of the COVID‐19 pandemic's impact on the oncological care pathway from January 2019 to December 2020. Our high‐volume, academic, tertiary center is a relevant example of the disruption caused by the pandemic throughout 2020. The region that it was conducted in one of the epicenters of the COVID‐19 outbreak in France and was severely hit during the second wave (Figure 1). Even if the cancer care pathway recovered to approximate pre‐pandemic activity levels, we did not clearly identify any compensatory phenomenon. Moreover, the impact of the second wave did not seem as substantial as the first except for medical announcement procedures, radiotherapy courses, and newly reviewed patient records in MTBMs.

All screening data for the Marne area over the years 2019 and 2020 were collected. Screening fell sharply during the first lockdown for both mammograms and FITs (up to 100% and 89.3% in April, respectively) which raised fears about delayed diagnoses, the appearance of more advanced diseases such as obstructive CRC, and shorten survival rates.^16^, ^17^, ^18^, ^19^, ^20^ For breast cancer screening activity, this deficit remained uncompensated for in the post‐lockdown months and saw an overall 19.5% decrease in 2020. CRC screening activity was overestimated (+25.3%) due to a national shortage of FIT supply in 2019.

Our findings about the impact of the first lockdown period commensurate with those reported worldwide for breast cancer care pathway and CRC in the USA and for FITs in the UK.^21^, ^22^ Nevertheless, these studies were published before the second wave and could not have assessed any possible subsequent compensatory effect. The absence of a rebound in activity suggests that cancer screening was heavily disrupted by healthcare systems faced with the pandemic and by patient's fears of contamination. Asymptomatic patients might have been severely impacted by the pandemic due to their asymptomatic setting and delayed diagnostic tests. Most countries were heavily impacted during the first wave, and did not catch up on the number of diagnoses during the second wave.^23^, ^24^, ^25^, ^26^ Stage migrations were observed which could impact prognosis. Healthcare systems reorganized and thus protected screening activity more efficiently which resulted in a limited second wave impact.

In April 2020, the number of announced cancer diagnoses and given personalized care plans were halved and oncogeriatric evaluations nearly halted (−86.4%). A small but non‐compensating upsurge was observed between lockdowns before a subsequent drop in October 2020 (−14.4%) with the approach of the second lockdown, corresponding to the decrease in new patient health records discussed in MTBMs (November 2020, −14.6%). In addition, the overall number of announced cancer diagnosis declined by 9.2% during 2020 compared to 2019, which was in sharp contrast with the consistent yearly growth rates observed during previous years but consistent with the 10.2% decline in pathology reports from a registry based study conducted in two states of the USA.^27^ A French multicentric study revealed a similar decline in new cancer cases between March and July 2020, however this did not apply to the whole year.^28^ On the contrary, histopathological analyses were roughly comparable between 2019 and 2020 (+2.0%) and rose every month during the pandemic resurgence. However, this did not affect the number of new cancer diagnosis announcements.

The number of new patient files discussing MTBMs decreased by more than a third in April (−35%) and again in May (−39.5%) which contrasted with the unusual rises seen (in spite of a resurgence of the pandemic) in June (+39.4%), September (+19.2%), October (+25%), and December 2020 (+31%) compared to the pre‐lockdown periods. Initial decreases and the struggle to catch up might be related to several factors such as a reduced screening activity, declining general practitioner and specialist availability, and decelerated sampling activity which all occurred due to the prioritization of COVID‐19 management plans. Lastly, the overall number of new files discussing MTBMs in 2020 was equal to that in 2019. These results are consistent with a Welsh study conducted in a tertiary care center.^29^ Even if the number of new diagnoses is comparable, major delays may have impacted prognoses and this impact will remain unknown for several more years.^17^, ^30^ However, as seen in other studies, diagnosis activity was maintained during the second lockdown.^17^, ^31^

A major gap between the new files and overall was noticed in June (18.7% vs. 39.4%), August (−2% vs. 16.5%), and October 2020 (11.5% vs. 25%). The upswings observed in new cases were highlighted for every primary site except for neuroendocrine tumors. This difference may have been related to delayed cancer diagnoses that should have been made during previous months. On the contrary, each MTBM experienced another unusual decline in November 2020 (−14.6%) except for bone, neurology, and breast malignancies. This could be interpreted as an impact of the resurgence of the COVID‐19 pandemic but at a lower intensity. Without reaching usual levels, increases in activity during the post‐lockdown period were described in a British study.^32^ As French COVID‐related health policy was national, no major‐specific adaptations were taken at a local level. We believe cancer care improved during the second wave due to a greater awareness of the entanglement between urgent and chronic care delivery associated with a better knowledge of COVID‐19 diagnosis, management, and prognosis.

During the first lockdown, medical oncology and radiotherapy were the least‐impacted treatment modalities (−12% and −15%, respectively) due to fewer resources being needed to provide efficient patient treatment compared to other procedures. They then remained stable over the following months and no impact was identified during the second wave except in October, 2020 (−16% for radiotherapy sessions). Oncological societies promoted the use of hypo‐fractionated radiotherapy as a way to maintain local tumor control without compromising patient safety.^33^ French national guidelines, after multidisciplinary discussion and in cases that do not constitute a loss of chances for patients, recommended a preference for the use of oral chemotherapy as a possible alternative whenever feasible.^34^ Recent large studies were able to establish a link between cancer and an increased risk of mortality following SARS‐Cov‐2 infection, but did not identify systemic cancer treatments as an independent risk factor, which may have encouraged a return to normal activity.^35^ However, we cannot exclude that delayed chemotherapy could have had an impact on tumor progression and survival depending on primary site and disease stage.^36^ This aspect must be properly assessed in future studies. Prognosis of patients with aggressive diseases might have been more severely impacted than others due to therapeutic delays and inadequate treatment strategy. Moreover, imposed therapeutic adjustments such as neoadjuvant treatments in aggressive resectable primary tumors due to delayed surgery were recommended with unknown consequences on prognosis.^37^

A drastic shift (April, −29.1% and May, −20.6%) in the number of oncological surgeries performed, interventional radiology (March, −50%; April −44.4%), and cytoreductive surgery with HIPEC (−100%) was observed. These results are consistent with the conclusions from several studies done throughout the world,^38^, ^39^ and may worsen the prognosis.^26^ Studies designed in Italy and the UK during the first wave found a respective 20% and 31% decrease in interventional radiology activity which was consistent with our findings.^40^, ^41^

No other study literature reported on interventional radiology activity data over the entire year 2020. Monthly oncological surgery activity rose or remained stable from July to December 2020 and did not seem to be impacted by the second lockdown. Anesthesiologists and ICUs are essential and limited resources during pandemics. Even if guidelines called for a prioritization of surgical interventions, delays in management might have impacted prognosis. A recent meta‐analysis showed that a 4‐week delay in surgery is associated with a 6%–8% increase in risk in all‐cause deaths for bladder, breast, colorectal, and lung cancer cases.^42^ Our results suggest that the dramatic consequences of the first peak were acknowledged since no more reductions were observed during the second lockdown. In contrast, our study highlights the impact that the pandemic had on cancer care activity in the year 2020. Another disruption has been predicted in periods following the pandemic as modeling studies suggest long‐term consequences. Impacts on colorectal screening and therefore, incidence could last until 2050.^20^ The impact of delayed diagnosis is also predictable.^30^ Moreover, these impacts may have massive medico‐economic consequences.^43^

There were several limitations in this study. No information was collected about patient demographic, medical or socioeconomic individual characteristics, disease stage, and survival rates. Neither supportive and palliative care nor clinical research activity were included in our data compilation. However, previously published study did not show any influence of age, gender, or distance between home and health care facility on care delivery during the epidemic.^37^ Dedicated studies might better specify the impact of the COVID‐19 pandemic on survival. However, predictions show that a substantial proportion of patients with curable tumors will progress to incurable disease.^23^, ^44^ An ongoing CAPANCOVID‐19 study is currently assessing the prognosis impact of the COVID‐19 pandemic patients with exocrine pancreatic cancer (https://clinicaltrials.gov/ct2/show/NCT04406571). Even if this study bears a monocentric setting, we believe that our facility broad activity can illustrate the upheaval that many cancer centers could have endured. The monocentric setting limits the external validity of this study and the analysis of the factors associated with the pandemic's disruption would have been interesting. However, we believe that our facility broad activity can illustrate the upheaval that many cancer centers could have endured. Moreover, our results are consistent with other multicentric studies and COVID‐19 burden does not seem proportional to the healthcare system disruption.^25^, ^28^, ^32^ Also, data collection was stopped in December 2020 at the onset of a pandemic resurgence which prevented us from analyzing the impact from a third wave. Finally, comparing 2020 activity with only 2019 without pooling data from previous years, may have altered the reliability of our usual activity data.

## 5 CONCLUSION

Disruptions in cancer care during the first COVID‐19 pandemic peak occurred due to the lack of healthcare system preparedness. The oncology care pathway returned to normal without catching up efficiently and was not similarly disturbed during the second lockdown. The extent of COVID‐19 vaccination rates, any herd immunity, medical knowledge accumulation, and public information campaigns might have normalized activity and prevented the COVID‐19 resurgence from impacting the oncology care pathway a second time. Unfortunately, previous pandemic peaks caused diagnosis and treatment delays with an unknown extent of damage. Consequences have only been estimated in model‐based analyses and thus further studies are needed in the future.

## AUTHOR CONTRIBUTIONS

Léonard Laurent: conceptualization, data curation, investigation, writing ‐ original draft, and writing ‐ review and editing. Mathias Brugel: conceptualization, data curation, formal analysis, investigation, methodology, validation, visualization, writing ‐ original draft, and writing ‐ review and editing. Claire Carlier: conceptualization, data curation, investigation, methodology, validation, visualization, writing ‐ original draft, and writing ‐ review and editing. Florentin Clere: investigation, writing ‐ review and editing. Aurélie Bertrand: investigation, writing ‐ review and editing. Damien Botsen: investigation, writing ‐ review and editing. Camille Boulagnon‐Rombi: investigation, writing ‐ review and editing. Véronique Dalstein: investigation, writing ‐ review and editing. Adeline Debreuve‐Theresette: formal analysis, investigation, writing ‐ review and editing. Sophie Deguelte: investigation writing ‐ review and editing. Christian Garbar: investigation, writing ‐ review and editing. Rachid Mahmoudi: investigation, writing ‐ review and editing. Antonin Marechal: investigation, writing ‐ review and editing. David Morland: investigation, writing ‐ review and editing. Jean‐Baptiste Rey: investigation, writing ‐ review and editing. Claire Schvartz: investigation, writing ‐ review and editing. Catherine Vallet: data curation, formal analysis, investigation, writing ‐ review and editing. Yacine Merrouche: supervision, writing ‐ review and editing. Florian Slimano: investigation, writing ‐ original draft, and writing ‐ review and editing. Olivier Bouché: conceptualization, data curation, investigation, methodology, project administration, resources, supervision, validation, visualization, writing ‐ original draft, and writing ‐ review and editing.

## CONFLICT OF INTEREST

Claire Carlier reported receiving honoraria as a speaker from Bristol Myers Squibb unrelated to this work. Damien Botsen reported receiving honoraria as a speaker and/or in an advisory role from Accord Healthcare, Amgen, Sanofi, Servier, and Pierre Fabre unrelated to this work. Florian Slimano reported receiving honoraria as a speaker and/or in an advisory role from Gilead, and Astra‐Zeneca unrelated to this work. Olivier Bouche reported receiving honoraria as a speaker and/or in an advisory role from Merck KGaA, Roche Genentech, Bayer, Astra‐Zeneca, Grunenthal, MSD, Amgen, Sanofi, Servier, and Pierre Fabre unrelated to this work. All other authors have no conflict of interest.

## Data Availability

N/A.
